# International Expert Consensus on Semantics of Multimodal Esophageal Cancer Treatment: Delphi Study

**DOI:** 10.1245/s10434-024-15367-w

**Published:** 2024-05-08

**Authors:** Charlène J. van der Zijden, Study Coordinators of the Erasmus MC Cancer Institute, Sjoerd M. Lagarde, Bianca Mostert, Joost J. M. E. Nuyttens, Manon C. W. Spaander, Bas P. L. Wijnhoven, For the International Expert Panel, Johanna W. van Sandick, Jolanda M. van Dieren, Francine E. M. Voncken, Jean-Pierre E. N. Pierie, Willem E. Fiets, Camiel Rosman, Peter D. Siersema, Heidi Rütten, Grard A. P. Nieuwenhuijzen, Geert-Jan Creemers, Erik J. Schoon, Maurice J. C. van der Sangen, Arjan Verschoor, Rutger Quispel, Meindert N. Sosef, Jeroen Buijsen, Hendrik H. Hartgrink, Marije Slingerland, Joos Heisterkamp, Laurens V. Beerepoot, Wouter L. Hazen, Tom Rozema, Karin Muller, Ewout A. Kouwenhoven, Simon Y. Law, Wendy W. Chan, Ian Y. Wong, Zhigang Li, Yin-Kai Chao, I-Chen Wu, Chiao-En Wu, Wing-Keen Yap, Seong Yong Park, Hiroya Takeuchi, Eisuke Booka, Ken Kato, Ito Yoshinori, Steven H. Lin, Guillaume Piessen, Anthony Turpin, Alexandre Taillez, Carlo Castoro, Alessandro Bastoni, Roberta Maselli, Marta Scorsetti, Thomas N. Walsh, Liam Grogan

**Affiliations:** 1 Department of Surgery, Erasmus MC Cancer Institute, Erasmus University Medical Center, Rotterdam, The Netherlands; 2 Department of Medical Oncology, Erasmus MC Cancer Institute, Rotterdam, The Netherlands; 3 Department of Radiotherapy, Erasmus MC Cancer Institute, Rotterdam, The Netherlands; 4 Department of Gastroenteroloy and Hepatology, Erasmus University Medical Center, Rotterdam, The Netherlands; 5 Department of Surgery, The Netherlands Cancer Institute - Antoni van Leeuwenhoek, Amsterdam, The Netherlands; 6 Department of Gastrointestinal Oncology, The Netherlands Cancer Institute - Antoni van Leeuwenhoek, Amsterdam, The Netherlands; 7 Department of Radiotherapy, The Netherlands Cancer Institute - Antoni van Leeuwenhoek, Amsterdam, The Netherlands; 8 Department of Surgery, Medical Center Leeuwarden, Leeuwarden, The Netherlands; 9 Department Medical Oncology, Medical Center Leeuwarden, Leeuwarden, The Netherlands; 10 Department of Surgery, Radboud University Medical Center, Nijmegen, The Netherlands; 11 Department of Gastroenterology, Radboud University Medical Center, Nijmegen, The Netherlands; 12 Department of Radiotherapy, Radboud University Medical Center, Nijmegen, The Netherlands; 13 Department of Surgery, Catharina Hospital, Eindhoven, The Netherlands; 14 Department of Medical Oncology, Catharina Hospital, Eindhoven, The Netherlands; 15 Department of Gastroenterology, Catharina Hospital, Eindhoven, The Netherlands; 16 Department of Radiotherapy, Catharina Hospital, Eindhoven, The Netherlands; 17 Department of Medical Oncology, Reinier de Graaf Group, Delft, The Netherlands; 18 Department of Gastroenterology, Reinier de Graaf Group, Delft, The Netherlands; 19 Department of Surgery, Zuyderland Medical Center, Heerlen, The Netherlands; 20 Department of Radiotherapy, Zuyderland Medical Center, Heerlen, The Netherlands; 21 Department of Surgery, Leiden University Medical Center, Leiden, The Netherlands; 22 Department of Medical Oncology, Leiden University Medical Center, Leiden, The Netherlands; 23 Department of Surgery, Elisabeth TweeSteden Hospital, Tilburg, The Netherlands; 24 Department of Medical Oncology, Elisabeth TweeSteden Hospital, Tilburg, The Netherlands; 25 Department of Gastroenterology, Elisabeth TweeSteden Hospital, Tilburg, The Netherlands; 26 Department of Radiotherapy, Verbeeten Instituut, Tilburg, The Netherlands; 27 Department of Radiotherapy, Radiotherapy Group, Apeldoorn, The Netherlands; 28 Department of Surgery, Zorggroep Twente, Almelo, The Netherlands; 29 Department of Surgery, School of Clinical Medicine, The University of Hong Kong, Hong Kong, China; 30 Department of Clinical Oncology, School of Clinical Medicine, The University of Hong Kong, Hong Kong, China; 31 Department of Surgery, Shanghai Chest Hospital, Shanghai, China; 32 Department of Surgery, Chang Gung Memorial Hospital-Linkou, Taipei, Taiwan; 33 Department of Gastroenterology, Kaohsiung Medical University Hospital, Kaohsiung Medical University, Kaohsiung, Taiwan; 34 Department of Hematology-Oncology, Department of Internal Medicine, Chang Gung Memorial Hospital at Linkou, Taoyuan, Taiwan; 35 Department of Radiotherapy, Chang Gung Memorial Hospital-Linkou, Taipei, Taiwan; 36 Department of Thoracic and Cardiovascular Surgery, Samsung Medical Center, Sungkyunkwan University School of Medicine, Seoul, Republic of Korea; 37 Department of Surgery, Hamamatsu University School of Medicine, Shizuoka, Japan; 38 Department of Head and Neck, Esophageal Medical Oncology, National Cancer Center Hospital, Tokyo, Japan; 39 Department of Radiotherapy, National Cancer Center Hospital, Tokyo, Japan; 40 Department of Thoracic Radiation Oncology, The University of Texas MD Anderson Cancer Center, Houston, TX USA; 41 Univ. Lille, CNRS, Inserm, CHU Lille, Cancer Heterogeneity Plasticity and Resistance to Therapies, Lille, France; 42 Department of Radiation Oncology, Oscar Lambret Center, Lille, France; 43 Department of Radiotherapy, Oscar Lambret Center, Lille, France; 44 Department of Surgery, Humanitas Clinical and Research Center - IRCCS, Rozzano, Milan, Italy; 45 Department of Medical Oncology, Humanitas Clinical and Research Center - IRCCS, Rozzano, Milan, Italy; 46 Department of Gastroenterology, Humanitas Clinical and Research Center - IRCCS, Rozzano, Milan, Italy; 47 Department of Biomedical Sciences, Humanitas Univerisity, Rozzano, Milan, Italy; 48 Department of Radiotherapy, Humanitas Research Hospital - IRCCS, Rozzano, Milan, Italy; 49 Department of Surgery, Connolly Hospital Blanchardstown, Dublin, Ireland; 50 Department of Medical Oncology, Connolly Hospital Blanchardstown, Dublin, Ireland

**Keywords:** Esophageal cancer, Neoadjuvant chemoradiotherapy, Definitive chemoradiotherapy, Active surveillance, Expert consensus, Delphi consensus study

## Abstract

**Background:**

Recent developments in esophageal cancer treatment, including studies exploring active surveillance following chemoradiotherapy, have led to a need for clear terminology and definitions regarding different multimodal treatment options.

**Objective:**

The aim of this study was to reach worldwide consensus on the definitions and semantics of multimodal esophageal cancer treatment.

**Methods:**

In total, 72 experts working in the field of multimodal esophageal cancer treatment were invited to participate in this Delphi study. The study comprised three Delphi surveys sent out by email and one online meeting. Input for the Delphi survey consisted of terminology obtained from a systematic literature search. Participants were asked to respond to open questions and to indicate whether they agreed or disagreed with different statements. Consensus was reached when there was ≥75% agreement among respondents.

**Results:**

Forty-nine of 72 invited experts (68.1%) participated in the first online Delphi survey, 45 (62.5%) in the second survey, 21 (46.7%) of 45 in the online meeting, and 39 (86.7%) of 45 in the final survey. Consensus on neoadjuvant and definitive chemoradiotherapy with or without surgery was reached for 27 of 31 items (87%). No consensus was reached on follow-up after treatment with definitive chemoradiotherapy.

**Conclusion(s):**

Consensus was reached on most statements regarding terminology and definitions of multimodal esophageal cancer treatment. Implementing uniform criteria facilitates comparison of studies and promotes international research collaborations.

**Supplementary Information:**

The online version contains supplementary material available at 10.1245/s10434-024-15367-w.

In Western countries, the standard treatment of locally advanced esophageal cancer consists of neoadjuvant chemo(radio)therapy (nCRT) followed by esophagectomy.^1^ Definitive chemoradiotherapy (dCRT) is an alternative treatment with an option for salvage esophagectomy in patients with residual or recurrent disease.^2^ Alternative treatment strategies are being explored that also try to minimize the role of surgery.^3–6^ Active surveillance after nCRT refers to an experimental approach in which patients with a complete response after nCRT are closely monitored for cancer regrowth instead of planned esophagectomy. Non-inferiority of overall survival at 2 years for active surveillance compared with planned surgery was demonstrated in the SANO trial.^7^

Given the complexity and evolving nature of esophageal cancer treatment, it is important to establish consensus on the definitions and semantics of the multimodal treatment modalities, including, but not limited to, active surveillance. For example, the term ‘salvage surgery’ is not uniformly defined in the literature and this limits the comparison of studies on dCRT.^8–11^ A unified terminology and conceptual framework are essential for precise communication, comparison and interpretation of outcomes, and future research collaboration. Organ-sparing treatments have already been implemented for prostate and rectal cancer, and consensus on outcome measures and definitions of terms used in clinical practice has been established.^12,13^

We conducted a Delphi study aimed at achieving expert consensus on the semantics of esophageal cancer treatment, including nCRT followed by resection or active surveillance and dCRT. The results of this study may help to frame further research and clinical practice guidelines on multimodal esophageal cancer treatment.

## Materials and Methods

This Delphi consensus study was conducted between June 2022 and June 2023. The Delphi method is a structured iterative survey-based technique to facilitate collective decision making and capture the breadth of expert opinions in a systematic manner.^14^ This study has not been submitted for review to the Medical Ethical Committee as it did not involve patients or privacy-sensitive data.

### Participants

Through email invitations, an international expert panel of surgeons, medical oncologists, gastroenterologists, and radiation oncologists were invited to participate in this study. All specialists practiced in a hospital providing active surveillance for esophageal cancer, and published studies on active surveillance. The study coordinators composed of two surgeons (BW, SL), one medical oncologist (BM), one gastroenterologist (MS), and one radiation oncologist (JN) from the Erasmus MC Cancer Institute. The international panel comprised a total of 49 participants representing three continents, including Europe, Asia, and the USA (Table 1).

**Table 1:** Characteristics of the participating experts

Characteristics	All experts [*n* = 49] (%)
Sex	
Male	40 (81.6)
Median age [IQR]	50 (42–55)
Continent	
Europe	36 (73.5)
Asia	12 (24.5)
USA	1 (2.0)
Specialty	
Surgical oncology	19 (38.8)
Medical oncology	11 (22.4)
Radiation oncology	10 (20.4)
Gastroenterology	9 (18.4)
Work experience	
≤10 years	15 (30.6)
>10 years	34 (69.4)

*IQR* interquartile range

### Literature Review

A systematic search was performed by an independent librarian in the Embase, Medline, Cochrane and Web of Science databases to identify commonly used terminology and definitions in esophageal cancer treatment (Online Resource Table S1).

Treatment terminologies for esophageal cancer were categorized into three groups: nCRT followed by surgery, nCRT followed by active surveillance, and dCRT with the possibility of subsequent resection.

### Delphi Survey Round 1 (June 2022)

Participants were requested to rate and comment on open-ended questions. They were given the opportunity to add terminology or leave comments for the coordinating group. Surveys were distributed via email using Castor Electronic Data Capture (EDC) version 2023.3.0.1 (Castor, Amsterdam, The Netherlands), and an automatic reminder was sent after a 2-week period to participants who had not responded.^15^ The survey was open for response for 12 weeks from the date of invitation and the results were discussed by the study coordinators of the Erasmus MC.

### Delphi Survey Round 2 (December 2022)

Participants were asked to express their agreement or disagreement with 33 statements in an online survey using Castor EDC. These statements were formulated by the study coordinators based on the findings from the initial Delphi survey round. The results of the first round were shared with the participants using text or images. Statements that fell beyond the study's scope, or did not align with the respondents' initial interest were not further questioned in the study. Participants had the opportunity to provide comments or add free text at the end of the second survey. The results of the second survey were then discussed with the study coordinators after a period of 8 weeks.

### Online Consensus Meeting (March 2023)

One week prior to the scheduled online consensus meeting, a list of items that reached consensus in both the first and second Delphi surveys was shared, as well as items on which no consensus was reached. The 1-h online meeting was hosted using Microsoft Teams Version 1.6.00 (Microsoft Corporation, Redmond, WA, USA). During this meeting, participants were presented with 10 additional statements or definitions pertaining to the treatment of esophageal cancer. A poll was utilized to gather the participants' agreement or disagreement on these statements, allowing for subsequent discussions among the participants. Following the conclusion of the meeting, the results were reviewed and discussed by the study coordinators.

### Delphi Survey Round 4 (May 2023)

A final online survey was conducted using Castor EDC. Participants were asked to express their agreement or disagreement on the remaining statements, which were formulated based on the outcomes and discussions from the online consensus meeting. The results of the previous round were shared with participants using text or images. The survey remained open for 8 weeks. Responses were discussed by the study coordinators with the aim to generate a final report.

### Statistical Analysis

Prior to start of the Delphi consensus study, the Erasmus MC panel established the definition of consensus as having an agreement threshold of ≥75%. This threshold aligns with the commonly reported median of 75% utilized in current literature as a widely accepted criterion for consensus.^16^ The statements and definitions that achieved consensus were analyzed using descriptive statistics and were presented as frequencies expressed in percentages (%).

## Results

### Literature View

The search yielded a total of 645 articles. Following a thorough evaluation of titles and abstracts (by researcher CZ), 101 articles were selected for extracting terminology as they discussed terminology relevant to esophageal cancer treatment. Articles that did not meet these criteria were excluded. The extracted terms were reviewed and supplemented with suggestions from the Erasmus MC panel. A final list of 38 terms was compiled, for which consensus was sought among the (inter)national participants.

### Participants

**Fig. 1:**
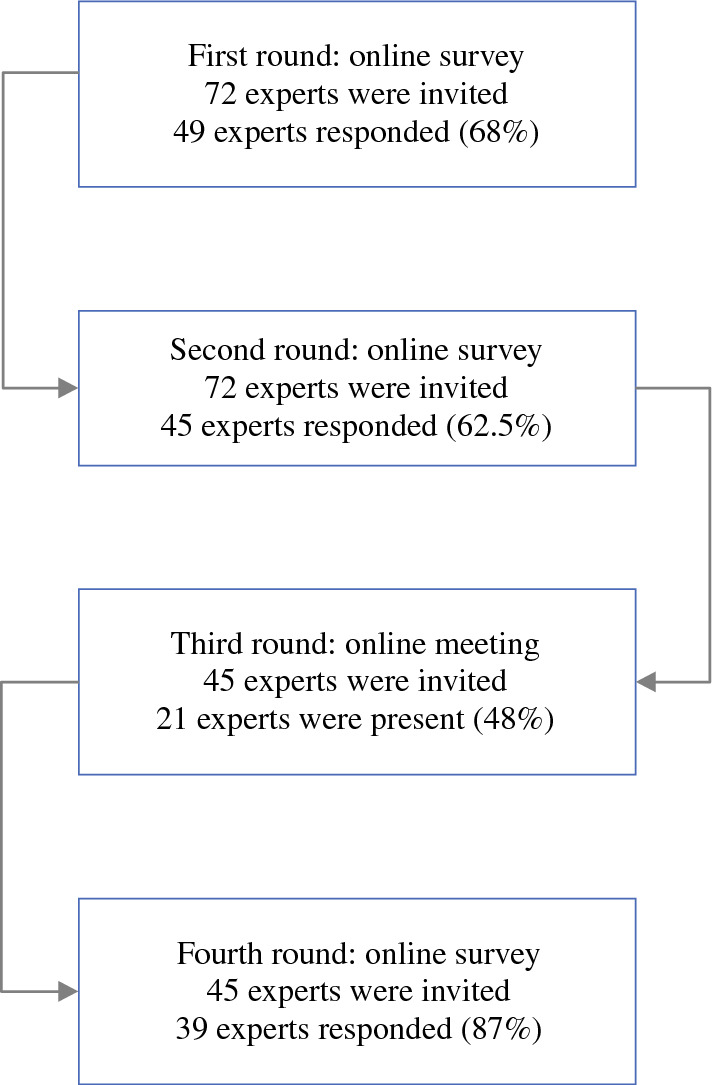
Overview of response to each Delphi round

A total of 72 national and international experts were invited to participate in the Delphi consensus study, of which 49 participated in the first round (response rate 68%) and 44 in the second round (response rate 61%). The 45 participants who responded to the first and second rounds were invited to the online consensus meeting (21 attended, response rate 47%), and 39 of 45 (87%) experts participated in the fourth round (Fig. 1).

An overview of the statements on which consensus was reached (87.1%) is shown in Table 2. The results for each Delphi round are shown in Online Resource Tables S2–5.

**Table 2:** An overview of the statements on which consensus was reached

Definitions		Consensus (%)
*Neoadjuvant chemoradiotherapy plus surgery*		
Main goal of nCRT	To downstage the tumor and to facilitate R0 resection, with the potential added benefit of treating micrometastases at an early stage	89
Chemotherapy regimen	A combination of platinum along with taxane, platinum in combination with a 5-fluorouracil derivate, or platinum combined with vinorelbine	91
Total dose of radiotherapy	41.4 Gy	96
Surgery after nCRT	Planned esophagectomy	76
*Neoadjuvant chemoradiotherapy followed by a strategy of observation*		
Strategy of observation after nCRT	Active surveillance	87
Main goal of active surveillance	To omit surgery and only perform surgery when loco(regional) tumor is detected without distant metastases	91
Evaluation of response to therapy	Response evaluation	97
First moment to start response evaluations	6–8 weeks after completion of nCRT	77
Diagnostic tests required during response evaluations	Yes	100
Diagnostic tests that should be performed during response evaluations	Diagnostic CT scan	76
	^18^F-FDG PET/CT scan	89
	Upper endoscopy with (bite-on-bite) biopsies	96
	Endoscopic ultrasonography with fine-needle aspiration of suspicious lymph nodes	91
	PET/CT combined with diagnostic CT scan	75
Surgery during active surveillance	Esophagectomy after surveillance	90
No tumor detected during active surveillance	Clinically complete response	82
If a tumor not (completely) responds to nCRT	Residual disease/cancer/tumor	78
Local disease	Tumor only located in the esophagus	96
Regional disease	Only tumor-positive lymph node(s) around the esophagus	98
Locoregional disease	Tumor in both the esophagus and surrounding lymph node(s)	98
Distant metastases	Organ metastases or non-regional lymph node metastases that occur after nCRT and during active surveillance, but outside the time window to surgery	97
Interval metastases	Organ metastases or non-regional lymph node metastases that occur after nCRT and during active surveillance, but within normal time window to surgery (without any delay)	95
*Definitive chemoradiotherapy (possibly followed by resection)*		
Main goal of dCRT	To cure esophageal cancer without surgery	96
Indications for treatment with dCRT	T4b carcinoma, proximal tumor, patients unfit for surgery and patients who refuse surgery	95
Chemotherapy regimen	A combination of a platinum along with a taxane, a platinum in combination with a 5-fluorouracil derivate, or a platinum combined with vinorelbine	82
Total dose of radiotherapy	50.4 Gy	91
Surgery after dCRT	Salvage esophagectomy	96

*nCRT* neoadjuvant chemoradiotherapy, *dCRT* definitive chemoradiotherapy, *Gy* Gray, ^*18*^*F-FDG PET/CT* 18F-fluorodeoxyglucose positron emission tomography/computed tomography

### Neoadjuvant Chemoradiotherapy Plus Surgery

The consensus among participants was that the main goal of nCRT is to downstage the tumor and to facilitate a radical (R0) resection, with the potential added benefit of treating micrometastases at an early stage. The recommended chemotherapy of the nCRT regimen was a combination of a platinum with a taxane, a platinum in combination with a 5-fluorouracil derivate, or a platinum combined with vinorelbine. Radiotherapy was defined as a total dose of 41.4 Gy. Surgery that is part of trimodality treatment and follows nCRT should be defined as *planned esophagectomy*.

### Neoadjuvant Chemoradiotherapy Followed by Observation

An observation strategy after achieving a clinical complete response following nCRT should be defined as *active surveillance*. The main goal of this strategy is to omit surgery in patients with a persistent complete remission or to postpone surgery until the presence of (loco)regional tumor is detected in the absence of distant metastases. Response to therapy can be assessed through *response evaluations*. Participants recommended planning the first response evaluation 6–8 weeks after the completion of nCRT. Recommended tests include a diagnostic computed tomography (CT) scan, 18F-fluorodeoxyglucose positron emission tomography (^18^F-FDG PET)/CT scan, ^18^F-FDG PET/CT combined with a diagnostic CT scan, upper endoscopy with (bite-on-bite) biopsies, and endoscopic ultrasonography with fine-needle aspiration of suspicious lymph nodes. The Delphi study did not delve into the sequence of these tests.

If no tumor is detected during active surveillance, it should be classified as a *clinically complete response*, while it should be called *residual disease/cancer/tumor* if a tumor did not (completely) respond to nCRT. If surgery is indicated during active surveillance (irrespective of the interval after nCRT), the consensus was to defined it as *esophagectomy after surveillance*.

### Disease Categories

Participants reached consensus on the definitions of different disease categories. *Local disease* refers to a tumor confined to the esophagus, while *regional disease* involves regional lymph nodes. *Locoregional disease* is characterized by the presence of tumor in both the esophagus and the regional lymph nodes. *Distant metastases* are defined as the presence of metastases in distant organs or non-regional lymph nodes that occur during active surveillance but outside the time-window to surgery. If metastases (distant) occur after nCRT but before the normal time window for surgery (i.e. 10–12 weeks after nCRT), they should be called *interval metastases*.

### Definitive Chemoradiotherapy (With or Without Esophagectomy)

The main goal of dCRT is to cure esophageal cancer without performing surgery. It was widely agreed that the indications for dCRT included T4b carcinoma, patients with cervical (proximal) esophageal tumors (distance from upper esophageal sphincter not further defined), patients who are unfit for surgery, and patients who refuse surgical intervention. The recommended dCRT regimen consists of a combination of a platinum with a taxane, a platinum in combination with a 5-fluorouracil derivate, or a platinum combined with vinorelbine, combined with a total dose of 50.4 Gy radiotherapy. When surgery is performed in the case of recurrent disease following dCRT, it should be called *salvage esophagectomy*.

No consensus was reached on the optimal approach for follow-up after dCRT in various clinical scenarios.

## Discussion

This international Delphi study led to a consensus on several aspects of esophageal cancer treatment. Consensus on statements was categorized according to different treatment modalities that focused on the main goal and regimen of therapy, evaluation of response to therapy, including diagnostic tests, and the definitions of different disease categories. Despite consensus being reached for 27 items (87%), the international expert panel could not agree on the optimal follow-up strategy after dCRT.

The consensus statements from this study do not represent the definite or sole truth. They offer a valuable and all-encompassing summary of the consensus reached. For example, consensus was reached on the need for diagnostic tests during response evaluations; however, the value of conducting an ^18^F-FDG PET/CT in combination with a diagnostic CT scan for each response evaluation (consensus 75%) is debatable. Some participants argued that a diagnostic CT scan is only of value when residual tumor has been confirmed and surgical resection is planned. On the contrary, the added value of a diagnostic CT scan can be questioned in patients with a complete response on the ^18^F-FDG PET/CT and endoscopy. We recommend to carefully consider its use given the associated costs and limited information when esophagectomy after surveillance is not indicated. Furthermore, new diagnostic tests may become available over time, potentially requiring a re-evaluation of these study findings. Variation in recommended diagnostic tests may also exist between countries due to costs/resources or because specific diagnostic tests are not yet available.

The efficacy and safety of active surveillance as an alternative to esophagectomy after nCRT has been shown at 2 years of follow-up and we are waiting for the long-term survival results.^4–7^ Smaller retrospective studies exploring the feasibility and safety of active surveillance after nCRT have shown similar outcomes.^17–21^ A recent systematic review of individual patient data also showed similar overall survival in patients who underwent active surveillance compared with patients with planned esophagectomy after nCRT.^22^ Considering these studies, and in anticipation of the long-term results of the SANO trial, we feel that active surveillance is an alternative treatment strategy that could be offered. This is supported by the outcome of a discrete choice experiment where patients were willing to accept a 16% lower 5-year overall survival rate if the possibility of esophagectomy after surveillance could be reduced to 30%.^23,24^ Even if active surveillance does not demonstrate to be equivalent overall survival after long-term follow-up results are known, it can remain a viable option for patients who prefer a non-surgical approach considering the associated risks of planned esophagectomy, including altered quality of life after surgery. Therefore, consensus on definitions and terminology as outlined in this Delphi study is needed.

The terminology used for surgery following a prolonged period after nCRT varies. Several studies refer to this as ‘salvage’ surgery.^25,26^ In alignment with the results of this Delphi study, the term 'salvage surgery’ should be used when esophagectomy is performed for recurrent disease after dCRT (radiation dose ≥50.4 Gy) where surgery is not part of the planned treatment regimen given a patient’s fitness or disease characteristics. When disease recurs, restaging should be performed, and only in a minority of patients may salvage esophagectomy be feasible and offer a chance for cure. This differs from esophagectomy within active surveillance after nCRT. Chemoradiotherapy dose differs and, as such, perioperative morbidity and mortality. In active surveillance, all patients should be eligible for surgery as the goal is not to prevent surgery but only to operate when cancer recurs. As the SANO trial shows, esophagectomy after surveillance is safe and results are similar to planned esophagectomy.^7^ The experts from this Delphi study therefore agreed that surgery performed after nCRT in the case of residual cancer detected during active surveillance should be referred to as 'esophagectomy after surveillance'. When esophagectomy follows nCRT, irrespective of response to treatment and outside an active surveillance strategy, it should be defined as 'planned esophagectomy'.

The large number of experts across three continents who actively participated is a strength of this study and increases its robustness and external validity.^27^ Second, the addition of an online consensus meeting between participants facilitated real-time interactions and dynamic discussions, enabling participants to engage in the exchanges of ideas, leading to quicker consensus building. Additionally, the use of virtual meeting tools enhanced flexibility, accommodating varied scheduled time zones and ensuring maximum participation from participants across the world.

There are a few limitations to consider. The study coordinators of the Erasmus MC made diligent efforts to invite international participants who were actively involved in providing active surveillance for esophageal cancer. We felt that these clinicians should be involved in this consensus document as they best understand the semantics of the multimodal treatment strategies for esophageal cancer. Nevertheless, only a few participants had a large experience with this treatment, which may have introduced some degree of selection bias. Efforts were made to invite participants from various regions and disciplines to ensure a diverse and representative panel. Second, while the consensus threshold of ≥75% aligns with commonly reported criteria, it is important to realize that this is a somewhat arbitrary threshold. Nevertheless, this threshold has been widely accepted in the literature and is consistent with other Delphi studies.

## Conclusion

This Delphi consensus study successfully attained a consensus among international experts on different facets of esophageal cancer treatment. The insights obtained from this study can promote the correct interpretation of study results and enhance the comparison of study results. It is hoped that consistent terminology will minimize misunderstandings, streamline data consolidation, and facilitate collaboration among healthcare providers from diverse geographical regions.

### Supplementary Information

Below is the link to the electronic supplementary material.

Supplementary file1 (DOCX 50 kb)
