# Supplementary material for: International Expert Consensus on Semantics of Multimodal Esophageal Cancer Treatment: Delphi Study
Source: Ann Surg Oncol. 2024 May 8;31(8):5075–82. doi: 10.1245/s10434-024-15367-w (PMC11236823; doi:10.1245/s10434-024-15367-w)
Supplement: Supplementary file 1 — Supplementary file1 (DOCX 50 kb) [file 10434_2024_15367_MOESM1_ESM.docx]

**International expert consensus on semantics of multimodal esophageal cancer treatment: a Delphi study**

Charlène J. van der Zijden, MD,^1^ study coordinators of the Erasmus MC Cancer Institute*, for the International expert panel^¥^

Study coordinators*

*Sjoerd M. Lagarde, MD, PhD,^1^ Bianca Mostert, MD, PhD,^2^ Joost J.M.E. Nuyttens, MD, PhD,^3^ Manon C.W. Spaander, MD, PhD,^4^ Bas P.L. Wijnhoven, MD, PhD^1^*

1. Department of Surgery, Erasmus MC Cancer Institute, Rotterdam, the Netherlands

2. Department of Medical Oncology, Erasmus MC Cancer Institute, Rotterdam, the Netherlands

3. Department of Radiotherapy, Erasmus MC Cancer Institute, Rotterdam, the Netherlands

4. Department of Gastroenteroloy and Hepatology, Erasmus University Medical Center, Rotterdam, the Netherlands

International expert panel^¥^

*Johanna W. van Sandick, MD, PhD,^5^ Jolanda M. van Dieren, MD, PhD,^6^ Francine E.M. Voncken, MD,^7^ Jean-Pierre E.N. Pierie, MD, PhD,^8^ Willem E. Fiets, MD, PhD,^9^ Camiel Rosman, MD, PhD,^10^ Peter D. Siersema, MD, PhD,^4,11^ Heidi Rütten, MD,^12^ Grard A.P. Nieuwenhuijzen, MD, PhD,^13^ Geert-Jan Creemers, MD, PhD,^14^ Erik J. Schoon, MD, PhD,^15^ Maurice J.C. van der Sangen, MD, PhD,^16^ Arjan Verschoor, MD, PhD,^17^ Rutger Quispel, MD, PhD,^18^ Meindert N. Sosef, MD, PhD,^19^ Jeroen Buijsen, MD, PhD,^20^ Hendrik H. Hartgrink, MD, PhD,^21^ Marije Slingerland, MD, PhD,^22^ Joos Heisterkamp, MD, PhD,^23^ Laurens V. Beerepoot, MD, PhD,^24^ Wouter L. Hazen, MD, PhD,^25^ Tom Rozema, MD,^26^ Karin Muller, MD, PhD,^27^ Ewout A. Kouwenhoven, MD, PhD,^28^ Simon Y. Law, MD, PhD, FRCESEd^29^ Wendy W. Chan, MD, PhD, MBBS, FRCR^30^ Ian Y. Wong, MD, PhD, MBBS, FRCSEd^29^ Zhigang Li, MD, PhD,^31^ Yin-Kai Chao, MD, PhD,^32^ I-Chen Wu, MD, PhD,^33^ Chiao-En Wu, MD, PhD,^34^ Wing-Keen Yap, MD, PhD,^35^ Seong Yong Park, MD, PhD,^36^ Hiroya Takeuchi, MD, PhD,^37^ Eisuke Booka, MD, PhD,^37^ Ken Kato, MD, PhD,^38^ Ito Yoshinori, MD, PhD,^39^ Steven H. Lin, MD, PhD,^40^ Guillaume Piessen, MD, PhD,^41^ Anthony Turpin, MD, PhD,^42^ Alexandre Taillez, MD, PhD,^43^ Carlo Castoro, MD, PhD,^44^ Alessandro Bastoni, MD, PhD,^45^ Roberta Maselli, MD, PhD,^46,47^ Marta Scorsetti, MD, PhD,^48^ Thomas N. Walsh, MD, PhD,^49^ Liam Grogan, MD, PhD,^50^*

5. Department of Surgery, The Netherlands Cancer Institute - Antoni van Leeuwenhoek, Amsterdam, the Netherlands

6. Department of Gastrointestinal Oncology, The Netherlands Cancer Institute - Antoni van Leeuwenhoek, Amsterdam, the Netherlands

7. Department of Radiotherapy, The Netherlands Cancer Institute - Antoni van Leeuwenhoek, Amsterdam, the Netherlands

8. Department of Surgery, Medical Center Leeuwarden, Leeuwarden, the Netherlands

9. Department Medical Oncology, Medical Center Leeuwarden, Leeuwarden, the Netherlands

10. Department of Surgery, Radboud University Medical Center, Nijmegen, the Netherlands

11. Department of Gastroenterology, Radboud University Medical Center, Nijmegen, the Netherlands

12. Department of Radiotherapy, Radboud University Medical Center, Nijmegen, the Netherlands

13. Department of Surgery, Catharina Hospital, Eindhoven, the Netherlands

14. Department of Medical Oncology, Catharina Hospital, Eindhoven, the Netherlands

15. Department of Gastroenterology, Catharina Hospital, Eindhoven, the Netherlands

16. Department of Radiotherapy, Catharina Hospital, Eindhoven, the Netherlands

17. Department of Medical Oncology, Reinier de Graaf Group, Delft, the Netherlands

18. Department of Gastroenterology, Reinier de Graaf Group, Delft, the Netherlands

19. Department of Surgery, Zuyderland Medical Center, Heerlen, the Netherlands

20. Department of Radiotherapy, Zuyderland Medical Center, Heerlen, the Netherlands

21. Department of Surgery, Leiden University Medical Center, Leiden, the Netherlands

22. Department of Medical Oncology, Leiden University Medical Center, Leiden, the Netherlands

23. Department of Surgery, Elisabeth TweeSteden Hospital, Tilburg, the Netherlands

24. Department of Medical Oncology, Elisabeth TweeSteden Hospital, Tilburg, the Netherlands

25. Department of Gastroenterology, Elisabeth TweeSteden Hospital, Tilburg, the Netherlands

26. Department of Radiotherapy, Verbeeten Instituut, Tilburg, the Netherlands

27. Department of Radiotherapy, Radiotherapy Group, Apeldoorn, the Netherlands

28. Department of Surgery, Zorggroep Twente, Almelo, the Netherlands

29. Department of Surgery, School of Clinical Medicine, The University of Hong Kong, Hong Kong, China

30. Department of Clinical Oncology, School of Clinical Medicine, The University of Hong Kong, Hong Kong, China

31. Department of Surgery, Shanghai Chest Hospital, Shanghai, China

32. Department of Surgery, Chang Gung Memorial Hospital-Linkou, Taipei, Taiwan

33. Department of Gastroenterology, Kaohsiung Medical University Hospital, Kaohsiung Medical University, Kaohsiung, Taiwan

34. Department of Hematology-Oncology, Department of Internal Medicine, Chang Gung Memorial Hospital at Linkou, Taoyuan, Taiwan

35. Department of Radiotherapy, Chang Gung Memorial Hospital-Linkou, Taipei, Taiwan

36. Department of Thoracic and Cardiovascular Surgery, Samsung Medical Center, Sungkyunkwan University School of Medicine, Seoul, Republic of Korea

37. Department of Surgery, Hamamatsu University School of Medicine, Shizuoka, Japan

38. Department of Head and Neck, Esophageal Medical Oncology, National Cancer Center Hospital, Tokyo, Japan

39. Department of Radiotherapy, National Cancer Center Hospital, Tokyo, Japan

40. Department of Thoracic Radiation Oncology, The University of Texas MD Anderson Cancer Center

41. Univ. Lille, CNRS, Inserm, CHU Lille, Cancer Heterogeneity Plasticity and Resistance to Therapies, Lille, France

42. Department of Radiation Oncology, Oscar Lambret Center, Lille, France

43. Department of Radiotherapy, Centre Oscar Lambret Center, Lille, France

44. Department of Surgery, Humanitas Clinical and Research Center - IRCCS, Rozzano, Milan, Italy

45. Department of Medical Oncology, Humanitas Clinical and Research Center - IRCCS, Rozzano, Milan, Italy

46. Department of Gastroenterology, Humanitas Clinical and Research Center - IRCCS, Rozzano, Milan, Italy

47. Department of Biomedical Sciences, Humanitas Univerisity, Rozzano, Milan, Italy

48. Department of Radiotherapy, Humanitas Research Hospital - IRCCS, Rozzano, Milan, Italy

49. Department of Surgery, Connolly Hospital Blanchardstown, Dublin, Ireland

50. Department of Medical Oncology, Connolly Hospital Blanchardstown, Dublin, Ireland

**Corresponding author:**

Charlène J. van der Zijden, Department of Surgery, Erasmus MC Cancer Institute, Erasmus University Medical Center, Dr. Molewaterplein 40, 3015GD Rotterdam, the Netherlands. Telephone: +31(0)107040704. Email: [c.vanderzijden@erasmusmc.nl](mailto:c.vanderzijden@erasmusmc.nl)

**Supplementary Materials - Index**

| **Supplementary Figures and Tables** |  |
| --- | --- |
| Table S1 | *pag. 4* |
| Table S2 | *pag. 5* |
| Table S3  Table S4  Table S5 | *pag. 9*  *pag. 12*  *pag. 14* |
|  |  |
|  |  |
|  |  |

**Supplementary Figures and Tables**

**Table S1**Search strategy

| **Embase**  ('esophagus tumor'/de OR 'esophagus cancer'/de OR 'esophagus carcinoma'/exp OR 'esophagogastric junction adenocarcinoma'/de OR 'esophagogastric junction cancer'/de OR (((esophag* OR oesophag* OR gastroesophag*) NEAR/3 (tumor* OR tumour* OR neoplas* OR cancer* OR malign* OR carcinom* OR adenocarcinom*))):ab,ti,kw) AND ('active surveillance'/de OR 'watchful waiting'/de OR 'conservative treatment'/mj/de OR 'organ preservation'/mj/de OR (((active) NEAR/3 (surveillance*)) OR ((watchful*) NEAR/3 (waiting*)) OR ((watch* OR see) NEAR/3 wait*)):ab,ti,kw OR (((conservative OR nonoperative* OR non-operative* OR nonsurgical* OR non-surgical* OR organ-sparing*) NEAR/3 (treatment* OR therap*)) OR ((organ OR esophag* OR oesophag* OR gastroesophag*) NEAR/3 (preservation*))):ti) AND [ENGLISH]/lim |
| --- |
| **MEDLINE Ovid**  (Esophageal Neoplasms/ OR (((esophag* OR oesophag* OR gastroesophag*) ADJ3 (tumor* OR tumour* OR neoplas* OR cancer* OR malign* OR carcinom* OR adenocarcinom*))).ab,ti,kw.) AND (Watchful Waiting/ OR * Conservative Treatment/ OR * Organ Preservation/ OR (((active) ADJ3 (surveillance*)) OR ((watchful*) ADJ3 (waiting*)) OR ((watch* OR see) ADJ3 wait*)).ab,ti,kw. OR (((conservative OR nonoperative* OR non-operative* OR nonsurgical* OR non-surgical* OR organ-sparing*) ADJ3 (treatment* OR therap*)) OR ((organ OR esophag* OR oesophag* OR gastroesophag*) ADJ3 (preservation*))).ti.) AND english.la. |
| **Cochrane CENTRAL**  ((((esophag* OR oesophag* OR gastroesophag*) NEAR/3 (tumor* OR tumour* OR neoplas* OR cancer* OR malign* OR carcinom* OR adenocarcinom*))):ab,ti) AND ((((active) NEAR/3 (surveillance*)) OR ((watchful*) NEAR/3 (waiting*)) OR ((watch* OR see) NEAR/3 wait*)):ab,ti OR (((conservative OR nonoperative* OR non NEXT operative* OR nonsurgical* OR non NEXT surgical* OR organ NEXT sparing*) NEAR/3 (treatment* OR therap*)) OR ((organ OR esophag* OR oesophag* OR gastroesophag*) NEAR/3 (preservation*))):ti) |
| **Web of Science**  TS=(((((esophag* OR oesophag* OR gastroesophag*) NEAR/2 (tumor* OR tumour* OR neoplas* OR cancer* OR malign* OR carcinom* OR adenocarcinom*)))) AND ((((active) NEAR/2 (surveillance*)) OR ((watchful*) NEAR/2 (waiting*)) OR ((watch* OR see) NEAR/2 wait*)) OR (((conservative OR nonoperative* OR non-operative* OR nonsurgical* OR non-surgical* OR organ-sparing*) NEAR/2 (treatment* OR therap*)) OR ((organ OR esophag* OR oesophag* OR gastroesophag*) NEAR/2 (preservation*))))) AND LA=(English) |

**Table S2**Answers to the first Delphi round

| *Neoadjuvant chemoradiotherapy* |  |
| --- | --- |
| **What is the main goal of nCRT?** | **n (%)** |
| To facilitate R0 resection | 10 (20.4) |
| To downstage the tumor | 7 (14.3) |
| To treat distant metastases | 0 (0) |
| To improve/increase survival | 2 (4.1) |
| Combination of answers | 30 (61.2) |
|  |  |
| **Which chemotherapeutic agents should be included?** | **n (%)** |
| Caboplatin + Paclitaxel | 27 (55.1) |
| Cisplatin + 5-FU | 5 (10.2) |
| Cisplatin + Paclitaxel | 3 (6.1) |
| Cisplatin + 5-FU + Docetaxel | 0 (0) |
| FOLFOX | 1 (2) |
| Combination of answers | 13 (26.5) |
|  |  |
| **What dose and fraction of radiotherapy is recommended?** | **n (%)** |
| 41.4 Gy (in 23 fractions) | 36 (73.5) |
| 50.4 Gy (in 28 fractions) | 3 (6.1) |
| Combination of answers | 10 (20.4) |
|  |  |
| **Which term do you prefer for surgery after nCRT?** | **n (%)** |
| Planned esophagectomy | 22 (44.9) |
| Standard esophagectomy | 16 (32.7) |
| Selective esophagectomy | 2 (4.1) |
| Combination of answers | 9 (18.4) |
|  |  |
| **How would you define a strategy of observation after nCRT?** | **n (%)** |
| Active surveillance | 27 (55.1) |
| Surveillance | 2 (4.1) |
| Watchful waiting | 2 (4.1) |
| Watch-and-wait | 2 (4.1) |
| Wait and see | 2 (4.1) |
| Surgery as needed | 1 (2) |
| Combination of answers | 13 (26.5) |
|  |  |
| **Which term do you prefer when surgery is performed during a strategy of observation?** | **n (%)** |
| Salvage esophagectomy/surgery | 20 (40.8) |
| Delayed esophagectomy/surgery | 11 (22.4) |
| Esophagectomy/surgery | 8 (16.3) |
| Postponed esophagectomy | 4 (8.2) |
| Combination of answers | 6 (12.2) |
|  |  |
| **Are diagnostic tests needed during observation after nCRT?*** | **n (%)** |
| Yes | 49 (100) |
| No | 0 (0) |
|  |  |
| **Which diagnostic tests are required during observation after nCRT?** | **n (%)** |
| Upper endoscopy | 1 (2.1) |
| Diagnostic CT-scan | 0 (0) |
| ^18^F-FDG PET/CT | 0 (0) |
| MRI-scan | 0 (0) |
| Combination of answers | 48 (97.6) |
|  |  |
| **In which time interval should these tests be repeated?** | **n (%)** |
| 12 weeks | 25 (51) |
| 8 weeks | 6 (12.2) |
| 6 weeks | 4 (8.2) |
| Other answers | 14 (28.6) |
|  |  |
| **Which term do you prefer for observation of response after nCRT?** | **n (%)** |
| Clinical response evaluation | 26 (53.1) |
| Response evaluation | 17 (34.7) |
| Serial clinical investigations | 4 (8.2) |
| Combination of answers | 2 (4.1) |
|  |  |
| **Which term do you prefer when no tumor is detected after nCRT?*** | **n (%)** |
| Clinically complete response | 40 (81.6) |
| Complete response | 4 (8.2) |
| Complete tumor response | 3 (6.1) |
| Other answers | 2 (4.1) |
|  |  |
| **Which term do you prefer for tumors that did not (completely) respond to nCRT?** | **n (%)** |
| Residual disease/cancer/tumor | 29 (59.2) |
| Incomplete response | 10 (20.4) |
| Combination of answers | 10 (20.4) |
|  |  |
| **Do you agree that local disease could be defined as tumor only located in the esophagus?*** | **n (%)** |
| Yes | 47 (95.9) |
| No | 2 (4.1) |
|  |  |
| **Do you agree that regional disease could be defined as tumor-positive lymph nodes?*** | **n (%)** |
| Yes | 48 (97.6) |
| No | 1 (2.1) |
|  |  |
| **Do you agree that locoregional disease could be defined as tumor in both the esophagus and surrounding lymph nodes?*** | **n (%)** |
| Yes | 48 (97.6) |
| No | 1 (2.1) |
|  |  |
| **Do you agree that distant disease could be defined as tumor in distant organs or non-regional lymph nodes?*^** | **n (%)** |
| Yes | 48 (97.6) |
| No | 1 (2.1) |
|  |  |
| **Which term do you prefer for disseminated disease after nCRT?** | **n (%)** |
| Distant metastases | 27 (55.1) |
| Interval metastases | 4 (8.2) |
| Progressive disease | 3 (6.1) |
| Combination of answers | 14 (28.6) |
|  |  |
| *Definitive chemoradiotherapy* |  |
| **What is the indication for dCRT?** | **n (%)** |
| To treat patients unfit for surgery | 3 (6.1) |
| An alternative for nCRT followed by surgery for better QoL | 2 (4.1) |
| To treat patients who refuse surgery | 0 (0) |
| To treat patients with T4b esophageal cancer | 0 (0) |
| Combination of answers | 44 (89.8) |
|  |  |
| **Do you agree that the main goal of dCRT is to cure esophageal cancer without surgery?*** | **n (%)** |
| Yes | 47 (95.9) |
| No | 2 (4.1) |
|  |  |
| **Which chemotherapeutic agents should be included?** | **n (%)** |
| Caboplatin + Paclitaxel | 24 (49) |
| Cisplatin + 5-FU | 5 (10.2) |
| Cisplatin + Paclitaxel | 3 (6.1) |
| Cisplatin + 5-FU + Docetaxel | 0 (0) |
| FOLFOX | 1 (2) |
| Combination of answers | 16 (32.7) |
|  |  |
| **What dose and fraction of radiotherapy is recommended?** |  |
| 50.4 Gy (in 28 fractions) | 27 (55.1) |
| Other answers | 11 (22.4) |
| Combination of answers | 11 (22.4) |
|  |  |
| **When would you use the term salvage esophagectomy?** | **n (%)** |
| For esophagectomy after dCRT (regardless of time interval between dCRT and resection and dosis of chemoradiotherapy) | 9 (18.4) |
| For esophagectomy after dCRT | 8 (16.3) |
| In case of surgery for localized disease recurrence after previous esophagectomy (e.g. recurrence at anastomotic site) | 2 (4.1) |
| Combination of answers | 30 (61.2) |
|  |  |
| **How would you define a strategy of observation after dCRT?** | **n (%)** |
| Active surveillance | 13 (26.5) |
| Surveillance | 13 (26.5) |
| Wait and see | 7 (14.3) |
| Watchful waiting | 2 (4.1) |
| Watch-and-wait | 1 (2.1) |
| Surgery as needed | 0 (0) |
| Other answers | 6 (12.2) |
| Combination of answers | 7 (14.3) |
|  |  |
| **Which term do you prefer for locoregional tumor after dCRT?** | **n (%)** |
| Residual disease/cancer/tumor | 24 (49) |
| Recurrent disease/cancer/tumor | 8 (16.3) |
| Regrowth of disease/cancer/tumor | 1 (2.1) |
| Combination of answers | 16 (32.7) |
|  |  |
| **Which term do you prefer for disseminated disease after dCRT?** | **n (%)** |
| Distant metastases | 26 (53.1) |
| Distant recurrence | 3 (6.1) |
| Interval metastases | 1 (2.1) |
| Progressive disease | 2 (4.1) |
| Combination of answers | 17 (34.7) |
|  |  |

*nCRT: neoadjuvant chemoradiotherapy; dCRT: definitive chemoradiotherapy; 5-FU: 5-fluorouracil; FOLFOX: Oxaliplatin, Leucovorin, 5-FU; Gy: Gray; ^18^F-FDG PET/CT: 18 Fluorodeoxyglucose Positron Emission Tomography/ Computer Tomography; MRI: magnetic resonance imaging.
*Consensus was reached
^Consensus was reached, but needs more clarification in other Delphi rounds*

**Table S3**Answers to the second Delphi round

| *Neoadjuvant chemoradiotherapy* |  |
| --- | --- |
| **Do you agree that the main goal of nCRT is to facilitate R0 resection, to downstage the tumor and to early treat micrometastases?*** | **n (%)** |
| Agree | 40 (88.9) |
| Disagree | 5 (11.1) |
|  |  |
| **Do you agree that in the setting of neoadjuvant chemoradiotherapy the following chemotherapeutic agents should be included: Platinum + Taxane or 5-FU (derivative) + Platinum or Platinum + Vinorelbine?*** | **n (%)** |
| Agree | 41 (91.1) |
| Disagree | 4 (8.9) |
|  |  |
| **Do you agree that 41.4 Gy is the standard radiotherapy dose in neoadjuvant chemoradiotherapy?*** | **n (%)** |
| Agree | 43 (95.6) |
| Disagree | 2 (4.4) |
|  |  |
| **Which term do you prefer for surgery after nCRT?** | **n (%)** |
| Planned esophagectomy | 31 (68.9) |
| Standard esophagectomy | 11 (24.4) |
| Other answers | 5 (11.1) |
|  |  |
| **How would you define a strategy of observation after nCRT in clinically complete responders?*** | **n (%)** |
| Active surveillance | 39 (86.7) |
| Watchful waiting | 5 (11.1) |
| Other answers | 1 (2.2) |
|  |  |
| **At what point would you start to perform response evaluations as part of a strategy of observation after nCRT?** | **n (%)** |
| 6 weeks | 22 (48.9) |
| 8 weeks | 10 (22.2) |
| 12 weeks | 13 (28.9) |
|  |  |
| **Do you agree that the main goal of an observation strategy is to omit surgery and only perform surgery when loco(regional) tumor is detected without distant metastases?*** | **n (%)** |
| Agree | 41 (91.1) |
| Disagree | 4 (8.9) |
|  |  |
| **Do you agree that a diagnostic CT-scan is required during observation after nCRT?*** | **n (%)** |
| Agree | 34 (75.6) |
| Disagree | 11 (24.4) |
|  |  |
| **Do you agree that a PET/low dose CT-scan is required during observation after nCRT?** | **n (%)** |
| Agree | 40 (88.9) |
| Disagree | 5 (11.1) |
|  |  |
| **Do you agree that an upper endoscopy with (bite-on-bite) biopsies is required during observation after nCRT?*** | **n (%)** |
| Agree | 43 (95.6) |
| Disagree | 2 (4.4) |
|  |  |
| **Do you agree that an endoscopic ultrasound with fine-needle aspiration of suspicious lymph nodes is required during observation after nCRT?*** | **n (%)** |
| Agree | 41 (91.1) |
| Disagree | 4 (8.9) |
|  |  |
| **In what time interval should these tests be repeated in the first year after completion of nCRT?*^** | **n (%)** |
| 8 weeks | 6 (13.3) |
| 12 weeks | 35 (77.8) |
| Other answers | 4 (8.9) |
|  |  |
| **Which term do you prefer for evaluation of response after nCRT?** | **n (%)** |
| Clinical response evaluation | 20 (44.4) |
| Response evaluation | 24 (53.3) |
| Other answers | 1 (2.2) |
|  |  |
| **Which term do you prefer for tumors that did not (completely) respond to nCRT?*** | **n (%)** |
| Residual disease/cancer/tumor | 35 (77.8) |
| Incomplete response | 10 (22.2) |
|  |  |
| **Which of the following terms do you prefer?** | **n (%)** |
| Residual disease | 19 (42.2) |
| Residual cancer | 9 (20) |
| Residual tumor | 7 (15.6) |
| Missing answer | 10 (22.2) |
|  |  |
| **Do you agree that metastases which occur after completion of nCRT and before surgery are called 'interval metastases'?*^** | **n (%)** |
| Agree | 37 (82.2) |
| Disagree | 8 (17.8) |
|  |  |
| **Do you agree that metastases which occur after nCRT but within an active surveillance strategy are called 'distant metastases'?*^** | **n (%)** |
| Agree | 36 (80) |
| Disagree | 9 (20) |
|  |  |
| *Definitive chemoradiotherapy* |  |
| **Do you agree that the indication for dCRT is to treat patients with T4b cancer, patients who are unfit for surgery or patients who refuse surgery?*^** | **n (%)** |
| Agree | 36 (80) |
| Disagree | 9 (20) |
|  |  |
| **Do you agree that in the definitive setting the following chemotherapeutic agents should be included: Platinum + Taxane or 5-FU (derivative) + Platinum or Platinum + Vinorelbine?*** | **n (%)** |
| Agree | 37 (82.2) |
| Disagree | 7 (15.6) |
| Missing answer | 1 (2.2) |
|  |  |
| **Do you agree that 50.4 Gy is the standard radiotherapy dose in dCRT?*** | **n (%)** |
| Agree | 41 (91.1) |
| Disagree | 3 (6.7) |
| Missing answer | 1 (2.2) |
|  |  |
| **Which term do you prefer for surgery after dCRT?*** | **n (%)** |
| Salvage esophagectomy | 43 (95.6) |
| Postponed esophagectomy | 1 (2.2) |
| Missing answer | 1 (2.2) |
|  |  |
| **How would you perform follow-up in patients after completion of dCRT?** | **n (%)** |
| Clinical follow-up | 11 (24.4) |
| Follow-up with imaging | 17 (37.8) |
| Follow-up on indication or in case of complaints | 8 (17.8) |
| Other answers | 8 (17.8) |
| Missing answer | 1 (2.2) |
|  |  |

*nCRT: neoadjuvant chemoradiotherapy; dCRT: definitive chemoradiotherapy; 5-FU: 5-fluorouracil; ^18^F-FDG PET/CT: 18 Fluorodeoxyglucose Positron Emission Tomography/ Computer Tomography.
*Consensus was reached
^Consensus was reached, but needs more clarification in other Delphi rounds*

**Table S4**Answers to the third Delphi round

| *Definitive chemoradiotherapy* |  |
| --- | --- |
| **We already agreed that the main goal of nCRT is to facilitate R0 resection, to downstage the tumor and to early treat micrometastases. Do you agree that we remove 'and to early treat micrometastases' as one of the main goals of nCRT?** | **n (%)** |
| Agree | 15 (71.4) |
| Disagree | 6 (28.6) |
|  |  |
| **Which term do you prefer for surgery after nCRT?*** | **n (%)** |
| Planned esophagectomy | 16 (76.2) |
| Standard esophagectomy | 5 (23.8) |
|  |  |
| **Which term do you prefer for surgery after nCRT and a strategy of observation (active surveillance)?*^** | **n (%)** |
| Postponed esophagectomy | 19 (90.5) |
| Delayed esophagectomy | 2 (9.5) |
|  |  |
| **At what time point would you start to perform response evaluations as part of a strategy of observation after nCRT?** | **n (%)** |
| 6 weeks | 7 (33.3) |
| 8 weeks | 10 (47.6) |
| 12 weeks | 4 (19) |
|  |  |
| **Would you like to perform a PET/low-dose CT-scan or a PET/CT combined with diagnostic CT-scan during response evaluations?*** | **n (%)** |
| PET/low-dose CT-scan | 5 (23.8) |
| PET/CT combined with diagnostic CT-scan | 16 (76.2) |
|  |  |
| **Which term do you prefer for evaluation of response after nCRT?** | **n (%)** |
| Clinical response evaluation | 14 (66.7) |
| Response evaluation | 7 (33.3) |
|  |  |
| **Do you agree that organ metastases or non-regional lymph node metastases which occur after nCRT and during active surveillance, but within normal time window to surgery (without any delay) are called interval metastases?*** | **n (%)** |
| Agree | 20 (95.2) |
| Disagree | 1 (4.7) |
|  |  |
| **Do you agree that organ metastases or non-regional lymph node metastases which occur after nCRT and during active surveillance but outside the time-window to surgery are called distant metastases?*^** | **n (%)** |
| Agree | 16 (76.2) |
| Disagree | 5 (23.8) |
|  |  |
| *Definitive chemoradiotherapy* |  |
| **Do you agree that the indication for dCRT is to treat patients with T4b cancer, patients who are unfit for surgery, patients who refuse surgery and patients with proximal esophageal tumors?*** | **n (%)** |
| Agree | 20 (95.2) |
| Disagree | 1 (4.7) |
|  |  |
| **How would you perform follow-up in patients after completion of dCRT?** | **n (%)** |
| Clinical follow-up | 3 (14.3) |
| Follow-up with imaging | 11 (52.4) |
| Follow-up only on indication or in case of complaints | 5 (23.8) |
| Other answer | 2 (9.5) |
|  |  |

*nCRT: neoadjuvant chemoradiotherapy; dCRT: definitive chemoradiotherapy; ^18^F-FDG PET/CT: 18 Fluorodeoxyglucose Positron Emission Tomography/ Computer Tomography.
*Consensus was reached
^Consensus was reached, but needs more clarification in other Delphi rounds*

**Table S5**Answers to the fourth Delphi round

| *Neoadjuvant chemoradiotherapy* |  |
| --- | --- |
| **Do you agree that an additional effect of nCRT might be to early treat micrometastases?*** | **n (%)** |
| Agree | 33 (84.6) |
| Disagree | 6 (15.4) |
|  |  |
| **Which term do you prefer for surgery after nCRT and a strategy of observation (e.g. active surveillance)?*** | **n (%)** |
| Interval esophagectomy | 4 (10.3) |
| Esophagectomy after surveillance | 35 (89.7) |
|  |  |
| **At what time point would you start to perform the first response evaluation as part of a strategy of observation after nCRT?*** | **n (%)** |
| 6-8 weeks | 30 (76.9) |
| 12 weeks | 9 (23.1) |
|  |  |
| **Do you agree that the term response evaluation can be used to evaluate the response after nCRT?*** | **n (%)** |
| Agree | 38 (97.4) |
| Disagree | 1 (2.6) |
|  |  |
| **Do you agree that organ metastases or non-regional lymph node metastases which occur after nCRT and during active surveillance, but outside the time-window to surgery are called distant metastases?*** | **n (%)** |
| Agree | 38 (97.4) |
| Disagree | 1 (2.6) |
|  |  |
| *Definitive chemoradiotherapy* |  |
| **How would you perform follow-up after completion of dCRT in patients who had cT4b tumor prior to start of therapy?** | **n (%)** |
| Clinical follow-up | 10 (25.6) |
| Follow-up with diagnostic tests | 19 (48.7) |
| Follow-up on indication or in case of complaints | 9 (23.1) |
| No follow-up | 0 (0) |
| Missing answer | 1 (2.6) |
|  |  |
| **How would you perform follow-up after completion of dCRT in patients who were unfit for surgery prior to start of therapy?** | **n (%)** |
| Clinical follow-up | 13 (33.3) |
| Follow-up with diagnostic tests | 11 (28.2) |
| Only if patients becomes operable/fit | 12 (30.8) |
| No follow-up | 2 (5.1) |
| Missing answer | 1 (2.6) |
|  |  |
| **How would you perform follow-up after completion of dCRT in patients who refused surgery prior to start of therapy?** | **n (%)** |
| Clinical follow-up | 13 (33.3) |
| Follow-up with diagnostic tests | 15 (38.5) |
| Follow-up on indication or in case of complaints | 10 (25.6) |
| No follow-up | 0 (0) |
| Missing answer | 1 (2.6) |
|  |  |
| **How would you perform follow-up after completion of dCRT in patients who had proximal esophageal cancer prior to start of therapy?** | **n (%)** |
| Clinical follow-up | 9 (23.1) |
| Follow-up with diagnostic tests | 18 (46.2) |
| Follow-up on indication or in case of complaints | 11 (28.2) |
| No follow-up | 0 (0) |
| Missing answer | 1 (2.6) |
|  |  |

*nCRT: neoadjuvant chemoradiotherapy; dCRT: definitive chemoradiotherapy.
*Consensus was reached*
